# Association of cord blood methylation fractions at imprinted insulin-like growth factor 2 (*IGF2*), plasma IGF2, and birth weight

**DOI:** 10.1007/s10552-012-9932-y

**Published:** 2012-03-06

**Authors:** Cathrine Hoyo, Kimberly Fortner, Amy P. Murtha, Joellen M. Schildkraut, Adelheid Soubry, Wendy Demark-Wahnefried, Randy L. Jirtle, Joanne Kurtzberg, Michele R. Forman, Francine Overcash, Zhiqing Huang, Susan K. Murphy

**Affiliations:** 1 Department of Community and Family Medicine, Duke University Medical Center, 2200 West Main Street, Ste. 600, Durham, NC 27705 USA; 2 Duke Cancer Institute, Duke University Medical Center, 6036 Hock Plaza, 2424 Erwin Rd, Durham, NC 27710 USA; 3 Division Maternal Fetal Medicine, Department of Obstetrics and Gynecology, Duke University Medical Center, 4022 Hosp South, Durham, NC 27710 USA; 4 Department of Behavioral Sciences, UT M.D. Anderson Cancer Center, 1515 Holcombe Blvd., Unit 1340, Houston, TX 77030-4009 USA; 5 Department of Radiation Oncology, Duke University Medical Center, 139 Env Safety Bldg., Durham, NC 27710 USA; 6 Pediatric Blood/Marrow Transplantation, Department of Pathology, Duke University Medical Center, 1430 N. Pavilion Bldg, Durham, NC 27705 USA; 7 Department of Epidemiology, UT M.D. Anderson Cancer Center, 1515 Holcombe Blvd., Unit 1340, Houston, TX 77030-4009 USA; 8 Division Gynecologic Oncology, Department of Obstetrics and Gynecology, Duke University Medical Center, 226 B Wing LSRC 450 Research Drive, Durham, NC 27708 USA; 9 Department of Obstetrics and Gynecology, Division of Clinical Epidemiology, School of Medicine, Duke University, 2200 West Main Street, Suite 600, Durham, NC 27705 USA; 10 Department of Pathology, Duke University Medical Center, Durham, NC 27708 USA

**Keywords:** *IGF2*, *H19*, ELISA, DNA methylation, Epigenetics, Epidemiology

## Abstract

**Purpose:**

Altered methylation at *Insulin*-*like Growth Factor 2 (IGF2)* regulatory regions has previously been associated with obesity, and several malignancies including colon, esophageal, and prostate adenocarcinomas, presumably via changes in expression and/or loss of imprinting, but the functional significance of these DNA methylation marks have not been demonstrated in humans. We examined associations among DNA methylation at *IGF2* differentially methylated regions (DMRs), circulating IGF2 protein concentrations in umbilical cord blood (UCB) and birth weight in newborns.

**Methods:**

Questionnaire data were obtained from 300 pregnant women recruited between 2005 and 2009. UCB DNA methylation was measured by bisulfite pyrosequencing*.* UCB plasma concentrations of soluble IGF2 were measured by ELISA assays. Generalized linear regression models were used to examine the relationship between DMR methylation and IGF2 levels.

**Results:**

Lower *IGF2* DMR methylation was associated with elevated plasma IGF2 protein concentrations (β = −9.87, *p* < 0.01); an association that was stronger in infants born to obese women (pre-pregnancy BMI > 30 kg/m^2^, β = −20.21, *p* < 0.0001). Elevated IGF2 concentrations were associated with higher birth weight (*p* < 0.0001) after adjusting for maternal race/ethnicity, pre-pregnancy BMI, cigarette smoking, gestational diabetes, and infant sex. These patterns of association were not apparent at the *H19* DMR.

**Conclusion:**

Our data suggest that variation in *IGF2* DMR methylation is an important mechanism by which circulating IGF2 concentrations, a putative risk factor for obesity and cancers of the colon, esophagus, and prostate, are modulated; associations that may depend on pre-pregnancy obesity.

## Introduction

The ‘developmental origins of adult disease susceptibility’ hypothesis posits that environmental exposures early in the development permanently alter the phenotype, influencing susceptibility to lifetime risk of obesity and several cancers [1]. These alterations are thought to be mechanistically driven by epigenetic alterations. The most stable and well-studied epigenetic mechanism is DNA methylation, which involves the covalent attachment of a methyl group to the 5-carbon position of the pyrimidine ring of cytosine residues when in 5′-CpG-3′ context. This epigenetic modification is faithfully transmitted to nascent DNA molecules during DNA replication and is maintained during somatic cell proliferation. DNA methylation contributes to the induction and maintenance of transcriptional gene silencing, propagating shifts in gene expression that orchestrate long-term susceptibility to several adenocarcinoma including colon [2, 3], ovary [4], prostate [5, 6], stomach [7], and esophagus [8]. Epigenetic mechanisms have also been hypothesized to link birth weight, a proxy for the well-being of the offspring in utero, with some of these cancers in adulthood [9–15]. However, genomic regions in which DNA methylation increases risk of these chronic diseases are still unknown. Ultimately, cancer prevention will require identification of aberrant epigenetic profiles driving risk, demonstration of their functional significance as well as environmental exposures associated with their perturbation and ‘windows’ of susceptibility during life’s course.

Identification of aberrant DNA methylation marks has been hampered by a lack of stable targets with known baseline levels, allowing for deviations from baseline to be detected. The need for epidemiologic studies to use specimens obtained from non-invasive means, such as peripheral blood leukocytes or buccal cells, which are often the only tissues available in population-based studies, has presented additional hurdles; epigenetic regulation and dysregulation can vary by tissue and cell type. Genomically imprinted genes are among several major classes of genes that are regulated by DNA methylation [16]. Unlike most autosomal genes that follow Mendelian laws of inheritance where both parentally derived alleles are expressed, imprinted genes are monoallelically expressed, with their functional haploidy controlled by parental allele-specific DNA methylation at differentially methylated regions (DMRs). Because these methylation marks are stable covalent modifications that are established before germ layer specification [17], these methylation profiles should be maintained in all germ layers, resulting in quantifiable systemic homogeneity in locus-specific epigenetic regulation [17, 18]. As such, imprinted DMRs stably exhibit ~50% methylation in somatic tissues.

*Insulin*-*like*-*growth factor*-*2* (*IGF2)* is perhaps the most intensively studied imprinted gene and is paternally expressed, with at least two regulatory DMRs located upstream of exon 3 and upstream of neighboring maternally expressed *H19* [19]. Hypermethylation (methylation fractions higher than the expected ~50%) at the *H19* DMR [2, 4] and CpG hypomethylation (methylation fractions lower than the expected ~50%) at the *IGF2* DMR [2] have been associated with higher *IGF2* mRNA levels suggesting increased transcriptional activity, presumably via relaxation of imprint controls. Such increased transcriptional activity is a common feature in adenocarcinomas of the ovary, esophagus and prostate [4, 8, 20–25]. *IGF2* DMR loss of methylation was observed in DNA that was obtained from peripheral blood leukocytes of adults exposed to severe caloric restriction periconeptionally, suggesting methylation shifts in response to adverse events occur early and are stable over many decades [26]. Based on these observations, methylation status at regulatory sequences of imprinted genes has been proposed as ‘archives’ or ‘biosensors’ of early exposure [27, 28]. However, the functional significance of these DMR methylation marks has not previously been demonstrated in epidemiologic studies. The objective of these analyses was to examine variation in DMR methylation at sequences regulating *IGF2/H19*, in relation to UCB plasma IGF2 protein concentrations. We also determined whether IGF2 protein concentrations are associated with variation in birth weight, an indicator of maternal and fetal well-being.

## Methods

### Study participants

A detailed description of study participants has been previously reported [29]. Briefly, between April 2005 and June 2009, 1101 eligible pregnant women seen at one of two Durham, North Carolina, obstetric facilities were identified through appointment logs and were invited to enroll in the Newborn Epigenetics STudy (NEST), a multiethnic cohort study. Approximately 85% (*n* = 940) provided written consent to participate. Eligibility criteria were as follows: age ≥18 years, English speaking, and an intent to use one of two Durham County obstetric facilities which enabled specimen collection at delivery. We excluded women infected with HIV and those planning to have their newborns adopted. Women were identified through appointment logs of two prenatal clinics serving the above two obstetrics care facilities: Duke maternal fetal medicine (MFM) and Durham Regional Hospital Obstetrics, Durham County, NC, as part of the NEST. Gestational ages at enrollment were 19–42 weeks (median = 28 weeks). This report is limited to 300 (32%) deliveries in whom DNA methylation analyses were performed and protein measurements are available. The 300 women are comparable to the 940 women with respect to maternal age (mean age 29 years), obesity (body mass index [BMI], 28 kg/m^2^), and sex of offspring (49% men). This study was approved by the Institutional Review Board at Duke University Medical Center.

### Data collection

#### Questionnaire data collection

A standardized questionnaire was either self-administered or completed with a trained interviewer. The questionnaire items included socio-demographic information including marital status, educational attainment, race/ethnicity, smoking history, and current smoking status, a history of common chronic diseases and conditions, dietary supplement use, weight and height before pregnancy (from which BMI and weight gain was computed). BMI was calculated as pre-pregnancy weight in kilograms divided by height in square meters (kg/m^2^), and weight gain was the difference between usual weight and weight at the delivery visit. At delivery, medical records were abstracted to verify cigarette smoking during pregnancy, reported morbidity and age, and to obtain additional parturition data including blood pressure and mode of delivery, body temperature and chorioamnionitis (the latter two as indicators of infection which may influence protein measurements), as well as offspring information including sex, anthropometric measurements, and 5-min Apgar scores.

#### Specimen collection and processing

Umbilical cord blood specimens were collected in vacutainer tubes containing EDTA within minutes of delivery and inverted gently to mix the anticoagulant with the blood. Specimens were stored in the hospital refrigerator until transported to the laboratory usually within 12 h of collection where they were centrifuged to isolate plasma and buffy coat, which was aliquotted and stored at −80°C. DNA was extracted from the white blood cells in the buffy coat using Puregene reagents according to the manufacturer’s protocol (Qiagen, Valencia, CA).

#### Methylation measurements

Description of methylation measurements and primers used has been described elsewhere [30]. Briefly, cord blood samples from the first 438 subjects were analyzed for methylation status at two DMRs controlling the expression of *IGF2*. The steps comprised modification of genomic DNA by treating it with sodium bisulfite using a high-throughput method previously described [4, 31] to convert unmethylated cytosines to uracils, leaving methylated cytosines unchanged. We then obtained a quantitative measure of methylation at each DMR using a Pyromark Q96 MD Pyrosequencing instrument (Qiagen; Valencia, CA). Three CpGs were measured at the *IGF2* DMR [2], located upstream of the imprinted *IGF2* promoter in exon 3, [2, 26] (chr 11p15.5; CpG site 1: 2,169,518; CpG site 2: 2,169,515; and CpG site 3: 2,169,499; NCBI Human Genome Build 37/hg19). We also measured DNA methylation at four CpG dinucleotides at the *H19* DMR at a sequence motif that binds the CTCF zinc-finger protein (chr 11p15.5; CpG site 1: 2,024,261, CpG site 2: 2,024,259, CpG site 3: 2,024,257, and CpG site 4: 2,024,254; NCBI Human Genome Build 37/hg19). Methylation data were 98% complete. We validated both pyrosequencing assays using defined mixtures of plasmids containing the fully methylated or unmethylated versions of the bisulfate-modified sequence.

#### Protein measurements

Plasma concentrations of IGF2 were measured using microplate enzyme-linked immunosorbent assays (Diagnostic Systems Laboratories, Webster, TX). Procedures for measurement followed the manufacture’s instructions. Absorbance was read using a microplate reader DSL, Webster, TX. The plasma IGF2 concentrations ranged from 50 to 2,000 ng/ml, and assay sensitivity was 2.2 ng/ml.

### Statistical analyses

For analyses, maternal age at enrollment was categorized as 18–30, 30–39 versus 40 years or older; marital status was dichotomized into married/living with partner versus not married; educational level was categorized into groups defined as up to high school, some college to college graduate versus graduate/professional school; race/ethnicity was categorized into African Americans versus non-Hispanic White versus other, cigarette smoking was dichotomized into smoking during pregnancy versus not smoking; birth weight was treated as both a continuous variable and dichotomized at less < 2500 versus ≥2500 g; maternal BMI was categorized into <25, 25–29, 30–34, and 35 + kg/m^2^; weight gain during pregnancy was dichotomized at <30 pounds versus ≥30 pounds; delivery mode was classified as vaginal vs. C-section; and histological evidence of chorioamnionitis was dichotomized as yes or no.

We examined the distribution of methylation fractions for normality at each CpG dinucleotide using Kolmogorov–Smirnov tests followed by principal components and confirmatory factor analyses to determine whether methylation at the three CpGs measured in the *IGF2* DMR and the four CpGs measured at the *H19* DMR were correlated to justify the use of a single mean for each DMR. Cronbach’s alpha values for CpGs were 86 and 87% at the *IGF2* DMR and *H19* DMR, respectively. We also examined the distributions of birth weight, IGF2 proteins, maternal weight gain and usual BMI for normality and found that with the exception of maternal BMI and weight gain, these variables were normally distributed among the 300 participants under study. Thus, subsequent analyses dichotomized maternal BMI at ≤30 kg/m^2^, and weight gain at the 75th percentile.

The main goal of our analyses was to determine whether DNA methylation fractions at each DMR were associated with IGF2 protein concentrations. Although race/ethnicity and adult BMI have been previously associated with IGF2 concentrations and will be considered potential confounders, F-tests were used to identify additional potential confounders, by identifying factors associated with statistically significant variation (*p* ≤ 0.20) in IGF2 protein concentrations, and methylation levels at each DMR. In addition, chorioamnionitis was examined for potential effect modification and confounding as previous studies of predictors of IGF2 protein in otherwise healthy adults suggest acute infection may affect protein concentrations [32]. Mode of delivery was evaluated for potential confounding or effect modifiers also because animal data suggests DNA methylation patterns may vary by delivery mode [33]. Because 75% of African Americans in this study were >30 kg/m^2^ compared to 25% of Whites, we fitted linear regression models to examine the relationship between IGF2 protein and DMR methylation for African Americans and Whites and also for obese and non-obese women, separately. To further examine the association *IGF2* DMR methylation and the protein product in the context of pre-pregnancy BMI or weight gain during the course of pregnancy, we repeated our analyses restricted to infants born to women >30 versus ≤30 kg/m^2^ and also gain weight below and above the 75th percentile. Cross-product terms for maternal BMI and DNA methylation at each DMR were also computed and included in statistical models where differences were observed in stratified analyses. Only variables with *p* value <0.05 were retained in final statistical models. To examine whether IGF2 protein concentrations were associated with birth weight, we also utilized linear regression models since birth weight was normally distributed. These latter models were adjusted for gestational diabetes, maternal BMI, race/ethnicity, and cigarette smoking, as these factors are known to independently influence birth weight in the general population. All statistical analyses were conducted in SAS version 9.3 (*SAS Institute, Cary, NC, USA*).

## Results

### IGF2 proteins and covariable

The average UCB plasma protein concentration of IGF2 among the newborns was 1029 ng/μl, standard deviation (SD = 458) (Table 1). Mean IGF2 concentration did not vary significantly by maternal age (*p* = 0.51), marital status (*p* = 0.82), smoking during pregnancy (*p* = 0.83), chorioamnionitis (*p* = 0.87) or indicators of maternal socioeconomic status such as educational attainment (*p* = 0.32) and type of health insurance coverage (*p* = 0.22) although five individuals with no insurance had significantly lower IGF2 levels. We observed lower IGF2 concentration in infants who were male (*p* value = 0.11), had low birth weight (*p* value = 0.12), those born by C-section (*p* value = 0.006) or to African American women (*p* value = 0.02). IGF2 concentrations were also lower in infants born to women with a pre-pregnancy BMI > 30 (*p* value = 0.06) and those for whom pregnancy weight gain was <30 lbs (*p* value = 0.09).

**Table 1:** Distribution of offspring *IGF2* concentrations by maternal and offspring characteristics

	IGF2 protein (ng/μl)				
	*n*	%	Mean	SD	*p* value
All participants	300	100	1028.78	457.75	–
*Maternal age (in years)*					
<30	166	56.1	1000.69	450.61	0.509
30–39	112	37.8	1043.15	452.31	
40+	18	6.1	1115.62	532.47	
Missing	4	1.3			
*Marital status*					
Married/partner	173	59.0	1027.44	451.68	0.815
Never Married/divorced/widowed/separated	120	41.0	1014.69	466.31	
Missing	7	2.3			
*Race*					
Black	156	54.2	963.52	435.62	
Caucasian	114	39.6	1131.22	465.39	0.020
Asian	5	1.7	1041.97	372.83	
Native American	2	0.7	1264.52	176.67	
Other	11	3.8	828.52	536.52	
Missing	12	4.0			
*Education*					
<HS/HSG/GED	117	39.0	991.76	477.33	
Some college/college graduate	175	58.3	1046.07	441.82	0.320
Missing	8	2.7			
*Insurance*					
Public	176	58.7	991.92	461.93	0.111
Private	114	38.0	1082.43	447.83	
No insurance	5	1.7	760.89	292.81	
Missing	5	1.7	–	–	
*Smoking*					
Non-smoker during peri-conception	244	81.3	1026.01	456.39	0.827
Smoker during peri-conception	56	18.7	1040.84	467.57	
*Birth weight*					
≤2,500(≤5 lb 8.2 oz)	43	14.3	922.61	476.02	0.121
>2,500 (>5 lb 8.2 oz)	249	83.0	1039.44	450.69	
Missing	8	2.7			
*Sex of offspring*					
Male	151	50.3	974.90	427.26	0.109
Female	143	47.7	1076.17	481.48	
Missing	6	2.0	–	–	
*Maternal BMI (kg/m* ^*2*^ *)*					
<25	133	44.3	1071.60	486.77	
25–29	57	19.0	974.44	409.15	
30–34	37	12.3	956.84	445.88	0.175
35+	39	13.0	919.73	346.13	
Missing	34	11.3	–	–	
*Weight gain during pregnancy*					
<30 lbs	197	65.7	989.65	429.97	0.088
>30 lbs	96	32.0	1086.81	504.30	
Missing	7	2.3	–	–	
*Delivery Route*					
Vaginal	170	56.7	1086.13	451.76	0.006
C-section	126	42.0	939.58	449.59	
Missing	4	1.3	–	–	
*Chorioamnionitis*					
Yes	22	7.3	1044.34	397.05	0.869
No	278	92.7	1027.55	462.82	

### *IGF2 *and *H19* DMR methylation and covariables

Table 2 shows the distribution of DNA methylation levels by maternal and infant characteristics that were associated with variation in IGF2 protein concentrations in Table 1. Overall, average methylation levels among the newborns were 48.5% (SD = 7.7) and 61.4% (SD = 7.7) at the *IGF2* and *H19* DMRs, respectively. At the *IGF2* DMR, methylation percentages were similar by race/ethnicity, sex, birth weight, delivery mode, weight gain during pregnancy, and BMI before pregnancy (*p* values > 0.20) (Table 2). However, we observed slight methylation differences in infants born to women with and those without histological evidence of chorioamnionitis at delivery (*p* value <0.18); chorioamnionitis was considered for confounding in the *IGF2* and *H19* DMR analyses as it exhibited significant methylation differences at *p* ≤ 0.20, as were race/ethnicity, birth weight and three correlated factors; maternal BMI, weight gain during pregnancy and mode of delivery.

**Table 2:** *IGF2* and *H19* DMR methylation fractions and maternal and offspring characteristics

	*IGF2* DMR % (sd)		*H19* DMR % (sd)	*p* value
All participants	48.5 (7.7)		61.4 (7.7)	–
*Race/ethnicity*				
African American	48.4 (9.5)		62.1 (8.3)	
White	48.2 (5.3)		60.7 (7.1)	
Other	50.0 (5.6)	*p* = 0.742	59.4 (7.0)	*p* = 0.199
*Sex of offspring*				
Male	48.4 (6.7)		61.7 (8.4)	
Female	48.3 (8.8)	*p* = 0.289	60.9 (7.1)	*p* = 0.478
*Birth weight*				
≤2,500(≤5 lb 8.2 oz)	48.6 (9.4)		59.8 (6.5)	
>2,500 (>5 lb 8.2 oz)	48.3 (7.5)	*p* = 0.875	61.5 (8.0)	*p* = 0.194
*Maternal BMI (kg/m* ^*2*^ *)*				
<25	48.8 (5.8)		60.7 (6.6)	
25–29	49.7 (10.2)		61.2 (7.4)	
30–34	49.3 (8.5)		63.0 (9.6)	
35+	47.5 (6.2)	*p* = 0.618	62.0 (9.1)	*p* = 0.390
*Weight gain during pregnancy*				
<30 lbs	48.7 (8.7)		61.9 (8.5)	
>30 lbs	47.9 (4.8)	*p* = 0.487	60.1 (6.0)	*p* = 0.079
*Chorioamnionitis*				
Yes	46.0 (7.0)		58.8 (5.9)	
No	48.7 (7.7)	*p* = 0.180	61.6 (7.8)	*p* = 0.128
*Delivery route*				
Vaginal	48.7 (8.9)		61.4 (7.7)	
C-section	48.2 (6.0)	*p* = 0.634	61.3 (7.9)	*p* = 0.950

### *H19* DMR methylation and IGF2 protein concentrations

Overall, we found a significant association between *H19* DMR methylation (β = −8.08, *p* = 0.02) and IGF2 protein concentrations (Table 3). However, adjusting for maternal BMI before pregnancy weakened this association (β = −4.69, *p* = 0.07) (data not shown), and additionally adjusting for chorioamnionitis and race/ethnicity further weakened this association (β = −3.64, *p* = 0.33). This association may be stronger in infants born to African Americans (β = −6.90, *p* = 0.15) than those to Whites (β = −2.54, *p* = 0.68), although neither reached statistical significance. However, BMI-specific analyses suggested this association may be more pronounced in infants born to non-obese women (β = −8.52, *p* = 0.09) compared to those born to obese women (β = 0.33, *p* = 0.95). Further adjustment for BMI, weight gain, and mode of delivery did not alter these findings. Analyses restricted to weight gain cutoff at the 75th percentile revealed no additional insights as the patterns of association between *H19* DMR methylation and IGF2 protein levels were similar to those of the analyses stratified by pre-pregnancy BMI (data not shown).

**Table 3:** Associations between methylation fractions at *IGF2* and *H19* DMRs and IGF2 circulating concentrations stratified by race and maternal obesity

	*IGF2* CpG methylation			*H19* CpG methylation		
	β	SE	*p* value	β	SE	*p* value
*All participants (n* = *300)*						
Crude	−9.87	3.90	0.01	−8.08	3.51	0.02
Adjusted^a^	−9.49	4.23	0.03	−3.64	3.72	0.33
*African Americans (n* = *156)*						
Crude	−7.94	4.23	0.06	−10.37	4.35	0.02
Adjusted	−8.89	4.70	0.06	−6.90	4.73	0.15
*Whites (n* = *114)*						
Crude	−12.28	9.36	0.19	0.19	6.35	0.98
Adjusted	−9.12	8.85	0.31	2.54	6.20	0.68
*BMI* < *30* *kg/m* ^*2*^ *(n* = *190)*						
Crude	−7.60	5.32	0.16	−8.52	5.03	0.09
Adjusted	−4.75	5.32	0.37	−7.67	5.08	0.13
*BMI* ≥ *30* *kg/m* ^*2*^ *(n* = *76)*						
Crude	−20.21	5.94	0.00	0.33	5.14	0.95
Adjusted	−20.41	6.51	0.00	1.36	5.38	0.80

^a^Models determining whether methylation at the *H19 *or *IGF2* DMRs predict protein levels. Models are adjusted in all participants, for birth weight, histological evidence of chorioamnionitis at delivery, maternal BMI before pregnancy and race. Models stratified by race/ethnicity and BMI were adjusted for birth weight, chorioamnionitis, and race/ethnicity or maternal BMI before pregnancy

### *IGF2* DMR methylation and IGF2 protein concentrations

Table 3 also shows that hypomethylation at the *IGF2* DMR was significantly associated with higher IGF2 protein concentrations (β = −9.87, *p* = 0.01). Adjusting for maternal BMI pre-pregnancy strengthened this association (β = −11.36, *p* = 0.01) and further adjusting for chorioamnionitis, birth weight and race/ethnicity did not alter this association. This association may be stronger in African Americans (*p* = 0.06) than Whites (*p* = 0.31). Analyses stratified by maternal BMI before pregnancy revealed an association between *IGF2* DMR methylation and IGF2 protein levels, which was considerably stronger in infants born to obese women (β = −20.41, *p* = 0.003) corresponding to ~110 ng/μl increase in IGF2 protein concentration for every 5% decrease in DNA methylation levels at the *IGF2* DMR. The magnitude of this association was markedly weaker in infants born to non-obese women (β = −4.75, *p* = 0.37). The cross-product term for obesity and *IGF2* DMR methylation, however, was not statistically significant (*p* = 0.78). Because weight gain during pregnancy is strongly associated with pre-pregnancy BMI, repeating these analyses within each stratum of pre-pregnancy weight gain also did not reveal any insight to clarify the relative importance of these factors as the associations were much stronger in the 71 infants born to women with the largest weight gain (>75th percentile), when compared to those who gained ≤75th percentile. Intriguingly, including maternal weight gain in models with pre-pregnancy BMI and its cross-product term did not suggest weight gain was an important factor in the association between *IGF2* DMR methylation and IGF2 protein concentration.

### IGF2 protein concentrations and birth weight

The mean birth weight in this population (*n* = 300) was 3.14 kg, with more than 95% of infants in the 2.2–5.2 kg range. We examined the association between IGF2 protein levels in UCB plasma and birth weight and found a linear increase in birth weight with increasing IGF2 protein levels (β = 0.28, SE = 0.07, *p* = 0.0003) (Table 4). This association was free from the influence of factors known to be associated with birth weight including maternal race/ethnicity, pre-pregnancy BMI, education, gestational diabetes, cigarette smoking, and sex of infant. Table 4 also shows that while maternal cigarette smoking was independently but inversely associated with birth weight (β = −230.2, *p* value = 0.009), pre-pregnancy BMI (β = 197.4, *p* value = 0.01) and Caucasian race/ethnicity (167.6, *p* value <0.0001) were positively associated with birth weight. Further inclusion of weight gain only widened confidence intervals, but did not alter risk estimates.

**Table 4:** Regression model β-coefficients and standard errors of the association between umbilical cord blood IGF2 protein concentrations and birth weight (*n* = 278)

Characteristic/factor	β	SE	*p* value
IGF2 protein concentrations (ng/μl)	0.275	0.075	0.0003
Race/ethnicity (African American vs. Caucasian)	167.6	39.57	<0.0001
Maternal cigarette smoking	−230.17	88.57	0.0099
BMI before pregnancy (obese vs. non-obese)	197.43	80.23	0.0145
Gestational diabetes	137.35	125.28	0.2739
Sex of infant	−50.52	67.77	0.4566

Sex, birth weight, or BMI could not be ascertained in 12 of 300 infants. Factors are mutually adjusted for each other. IGF2 protein levels are associated with higher birth weight (*p* = 0.0003); association is independent of race/ethnicity, maternal cigarette smoking, pre-pregnancy BMI, gestational diabetes, and sex

## Discussion

We examined DNA methylation within two regulatory regions of imprinted *IGF2* in relation to circulating IGF2 concentrations in UCB of newborns. We observed a significant increase in IGF2 protein concentrations with decrease in methylation levels at the *IGF2* DMR. This association was strongest among infants born to women with a pre-pregnancy BMI > 30, similar in African Americans and Caucasians. Effect sizes of a similar magnitude have been previously reported at the *IGF2* DMR and other regions [26, 34, 35]. We also evaluated IGF2 protein concentrations in relation to birth weight, and observed elevated IGF2 protein concentrations were associated with a higher birth weight. We found no evidence for associations between CpG methylation at the *H19* DMR and IGF2 protein concentration after adjusting for maternal obesity and race/ethnicity. We provide the first evidence for an association between DNA methylation at the *IGF2* DMR and IGF2 protein concentrations. These findings are consistent with the interpretation that DNA methylation at the *IGF2* DMR is functionally relevant to the production of the potent mitogenic growth factor IGF2 and that soluble IGF2 independently contributes to variation in birth weight.

The exact mechanism by which loss of methyl groups at the I*GF2* DMR increases IGF2 protein concentrations, exacerbated by maternal obesity, is still unknown, although changes in DNA methylation on the maternally derived allele has been shown to relax imprint controls, reactivating the otherwise silent maternally derived allele, which has been shown in previous studies to increase *IGF2* mRNA and presumably protein products. Because imprinted genes are usually found in clusters [36] and their regulation may be networked [37], methylation alterations of this DMR have the potential for regulating (and dysregulating) multiple genes on different chromosomes. Therefore, methylation at this single DMR can provide information on epigenetic dysregulation elsewhere in the genome [18]. Using DNA obtained from leukocytes, methylation differences of a similar magnitude were reported among Gambians [17] and Dutch [26] adults who were exposed to nutritional challenges periconceptionally. Together, these findings support the idea that methylation marks acquired in the periconceptional period is similar across tissue types and is stable over many years. The stability of DNA methylation marks at the *IGF2* DMR during a 3-year period was also recently reported among adult population controls [38]. The small magnitude of differences is also consistent with the contention that methylation marks acquired as a result of adjustments to perceived environment may not be as large as those found in cancer [39, 40]). Strong inverse associations between DNA methylation levels at the *IGF2* DMR using DNA obtained from leukocytes, biallelic expression of this otherwise monoallelically expressed gene, and elevated colon cancer risk [2, 38, 41] have been reported. In tissue specimens, hypermethylation at the *H19* DMR is also frequent in cancers of the ovary [42], breast [43], esophagus [44], and prostate [45]. The *IGF2* and *H19* DMRs have been previously proposed as biomarkers, biosensors, or archives of early exposures [2, 27, 28]. Therefore, our findings of a strong relationship between lower methylation at the *IGF2* DMR and elevated IGF2 protein levels support the use of DNA methylation levels at this and perhaps other DMRs as biosensors or archives of early exposure. Differences observed by maternal obesity may be partly explained by genetic factors that also play a role in modulating IGF2 levels in the offspring.

Findings that protein levels of the mitogenic IGF2 are strongly associated with decreased methylation at the *IGF2* DMR and with birth weight support the interpretation that variation in birth weight is modulated, at least in part, by *IGF2* plasticity. This may also be occurring at other DMRs, if hypothesized co-regulation with other imprinted regions [37] can be demonstrated. Previous studies have reported an association between circulating IGF2 protein concentrations and high birth weight [46] as well as risk of obesity [47] and several cancers [48, 49] in adulthood, suggesting early exposures may contribute to the variation in birth weight via epigenetic alterations.

Our inability to find a consistent association between *H19* DMR methylation and IGF2 protein levels except among the 188 infants born to women with BMI < 30 was unexpected. Current thinking for transcriptional control of the imprinted *IGF2/H19* domain correlates increased DNA methylation at the *H19* DMR with failure to bind methylation sensitive CTCF, which can transcriptionally activate *IGF2* on the normally silent maternal chromosome [50–52]. We cannot exclude the possibility that these were chance findings, since the statistical power to conduct multivariable analyses may have been inadequate. However, others have also failed to find a relationship between methylation at the *H19* DMR and *IGF2* transcription among children conceived in vitro and in vivo [52] suggesting that at the *H19* DMR, other processes including genetic factors or other unmeasured environmental stimuli may contribute to increased IGF2 protein concentrations and body size, independent of DNA methylation alterations.

The main limitation of this study is the small sample size which, although more than adequate for examining crude relationships among DMR methylation fractions, protein IGF2 concentrations and birth weight, may have been inadequate for multivariable analyses, especially when testing interaction by race and maternal BMI. Despite the need for larger studies to clarify these relationships, the analyses raised the possibility that maternal obesity, which may be driven by other factors including genetic variation, also plays a role in modulating IGF2 concentrations. This suggests that a more detailed understanding of these epigenetic targets will require genetic analyses within the sequences under evaluation. Another limitation is that DNA obtained from unfractionated blood containing multiple cell types may have distinct epigenetic profiles depending on the cell population analyzed. However, we have previously described the analysis of fractionated cord blood for methylation at the same *H19* and *IGF2* DMRs, and the major cell types showed no significant differences in methylation levels [30]. Additionally, overall white blood cell counts vary and could confound the methylation fraction measures. However, inclusion of chorioamnionitis during parturition into statistical models did not alter our findings, suggesting variation in total cell counts may not contribute to differences in methylation at these DMRs. Furthermore, although we demonstrated an association between DMR methylation and protein levels downstream, we were limited by a lack of transcription data. However, a positive correlation between the levels of *IGF2* mRNA and DNA methylation at these DMRs has been previously reported. These limitations notwithstanding, our findings suggest that *IGF2* DMR plasticity is an important mechanism by which IGF2 protein levels are modulated.

In summary, among 300 newborns, we found a strong inverse association between DNA methylation at the *IGF2* regulatory sequence and elevated plasma protein concentrations of the encoded mitogenic growth factor. This relationship appeared to be stronger in infants born to women who were obese before pregnancy, but not weight gain. Higher IGF2 protein concentrations were also associated with higher birth weight. Herein, we provide evidence in support of the functional significance of aberrant methylation profiles at regulatory elements controlling the expression of imprinted *IGF2*, supporting the use of *IGF2* DMR methylation as an archive or biosensor of early exposure. If co-regulation of imprinted domains can be demonstrated in humans, and transcripts are also associated with DNA methylation and protein levels for other imprinted genes in the same population, our findings would support the use of methylation marks in an imprint regulatory network to improve exposure assessment in studies of cancer and other chronic diseases.
